# Elder Abuse and Neglect Versus Parricide

**DOI:** 10.5812/ijhrba.14983

**Published:** 2013-12-25

**Authors:** Sergei V Jargin

**Affiliations:** 1 Department of Public Health, Peoples’ Friendship University of Russia, Moscow, Russia

**Keywords:** Elder Abuse, Parricide, Alcoholism

## Abstract

Violence in families and elder abuse can take many forms, which his sometimes difficult to recognize. In Russia, elder abuse is rarely discussed in professional literature and mass media. A border between elder abuse and parricide can be indistinct. Borderline cases can include involvement of older people in binge drinking, denial of help, and manipulation towards suicide. Three example cases are discussed in this report. A concluding point is that for prevention of parricide, it should be kept devoid of its reputation as an extraordinary crime, committed mainly by mentally ill individuals. The perpetrators can be mentally healthy or have a personality disorder. Parricide can have trivial appearance, not always recognized as such by victims and social environment.

## 1. Introduction

Elder abuse can have many forms; it is generally under-recognized and under-reported (1). Physicians under-report elder abuse because of their unfamiliarity with legislation or the belief that they may not have appropriate evaluation skills (2). Victims often exhibit low self-esteem, blame themselves for the abuse, do not want to admit their vulnerability, or ‘to betray’ their families (2). The theme is “shrouded in silence, stigma, and shame” (3). Factors associated with elder abuse include advanced age, low income, functional impairment, drug abuse, and lack of social support (4). Violence may take many forms, often being subtle and insidious (5). A border between parricide and elder abuse, resulting in parent's death, can be indistinct. Parricide is sometimes considered as a violent crime committed predominantly by mentally ill individuals (6), often involving excessive amount of destructive violence (7). According to (8), most parricides belong to two main categories. Adolescent parricides tend to be cataclysmic reactions to enduring, severe physical abuse, perpetrated by an individual who is typically neither conduct disordered nor psychotic. Adult parricides tend to be tragic conclusions of highly conflictual relationships between untreated psychotic individuals and their parents. The typical profile of an adult perpetrator was described as a young single unemployed male, living with his victim, and suffering from schizophrenia with comorbidity of alcohol or drug abuse (9). Among adult perpetrators, schizophrenia with symptoms of psychosis present at the moment of the parricide, was the most common diagnosis (7).

Although it is difficult to generalize having no reliable statistics, being acquainted with some cases and the permissive atmosphere, it should be stressed that parricide can be a tactics, conducted consciously or in part subconsciously by mentally healthy perpetrators aimed at elimination of an elderly parent. It can include involvement into binge drinking, denial of help, and manipulation towards self-destructive behavior up to a suicide. Such cases can be hardly distinguishable from elder neglect and abuse, or assisted suicide. One of the most frequent motives is economic issues (6), in Russia, particularly, appropriation of apartments and houses. Cases with a purely economic motivation would be obviously unrelated to Oedipus complex, described as a psychodynamic basis of the patricide (10, 11). Moreover, high prevalence of mental diseases, found among those who commit parricide, can be partly explained by the fact that such cases were looked for psychiatric institutions (12); on the other hand, crime committed by mentally healthy individuals probably more often remains undisclosed. The cases described below were reported to the authorities while the efforts were made to prevent identification of persons. At the same time, former perpetrators and some other involved individuals were helpful in preparation of this report, participated in clarification of motives, and psychological backgrounds.

## 2. Case 1

It is understandable that a young man would like to have his own apartment; and his mother, divorcing her alcoholic husband, would try to secure the apartment for her son. A large apartment was exchanged for 2 smaller flats, one for the mother and grandmother, and one for the father and son. According to the Soviet-time registration system, if a person registered alone in a state-owned apartment, it was not inherited by relatives but taken by the state. However, if somebody else had been registered in the same apartment, it usually remained at his or her disposal (if a considerable excess of dwelling space resulted, some other people could be settled in). The father, an alcohol abuser, had been a good engineer but dementia symptoms had become recognizable during the last 3-4 years. At the age of about 54 years, he was unemployed but intended to start working at a workshop for disabled. His son was registered together with the father but lived with the mother and arranged drinking parties in his father’s apartment, who participated in the binge drinking. The son informed his former schoolmate Sergei that he had been repeatedly speaking with his father about hopelessness of his condition during the parties and thereafter, saying that the dementia would only worsen and his life had no sense anymore, and that they had together decided to commit suicide as a solution. Then, he invited Sergei to participate, his father had agreed to commit suicide, and they would just help if necessary. They came in the evening, drank some vodka, and another bottle was left for the next morning. In the morning, after the bottle had been finished, the father was accompanied to a hook in the wall, with a sling on his neck. The case was treated by the authorities as a suicide. Irreversibility of dementia and mercy as a motive were discussed by the perpetrators. Despite the fact, alcohol-related dementia could be -at least in part- reversible with abstinence (13, 14). The son never had any symptoms of psychosis but demonstrated personality traits classified by the author of this paper as schizoid and sadistic: inclination to elaborated reasoning including the idea of murder; in his childhood, he mistreated his grandmother, apparently with an ethnic motive. The grandmother had married a person of non-Russian ethnicity, which supposedly had a negative impact on the grandson’s life. As for Sergei, his motives were juvenile curiosity and immediate perspective of alcohol consumption offered by the accomplice. Sergei maintained that he had not believed until the end that something serious would indeed happen. However, after the father’s death, his former schoolmate gave him his apartment key now and then, a subconscious anticipation, which could have been a motive.

## 3. Case 2

This case shows what can happen to an elderly apartment owner in Russia. In the past, Emma worked at a Soviet trade mission abroad. Personnel of the mission were offered an opportunity to purchase apartments or rooms in a new building in Moscow. There were only 3-room apartments in that building; Emma paid for 2 rooms, and a colleague, who remained single until her death, bought the third. Later on, the building was expropriated by the state, while owners received certain amounts in Rubles. Emma had adopted her orphan niece; later the stepdaughter moved with her family to another place, while Emma remained alone in her two rooms. After the death of neighbor, a 3-person family was settled by the authorities into the third room. One year thereafter, Emma died and the young family occupied the whole apartment. At that time, Emma was 73 years old with no diagnosed serious disease, apart from an enlarging hernia in a post-hysterectomy scar. There was no visible evidence of unnatural death and neither forensic examination nor autopsy was performed. The case can be seen as a cynical act on the part of the authorities who settled a young family into one room of the apartment, where two other rooms were occupied by an elderly person, obviously in anticipation of her death. It can be also seen as elder neglect on the part of stepdaughter who had not visited Emma until the last day of her life (which can be explained by the fact that Emma had previously mistreated her stepdaughter). The reason for reporting this case, on the background of a variable attitude to private property in Russia, is that neighbors and other persons are duly informed that young people among them may learn that such events can pass without consequences. The permissible atmosphere is maintained by similar cases, as well as overt crime for misappropriation of apartments and houses, which is known to happen in Russia and would increase if not exposed and no appropriate measures would be taken.

## 4. Case 3

**Figure 1.**
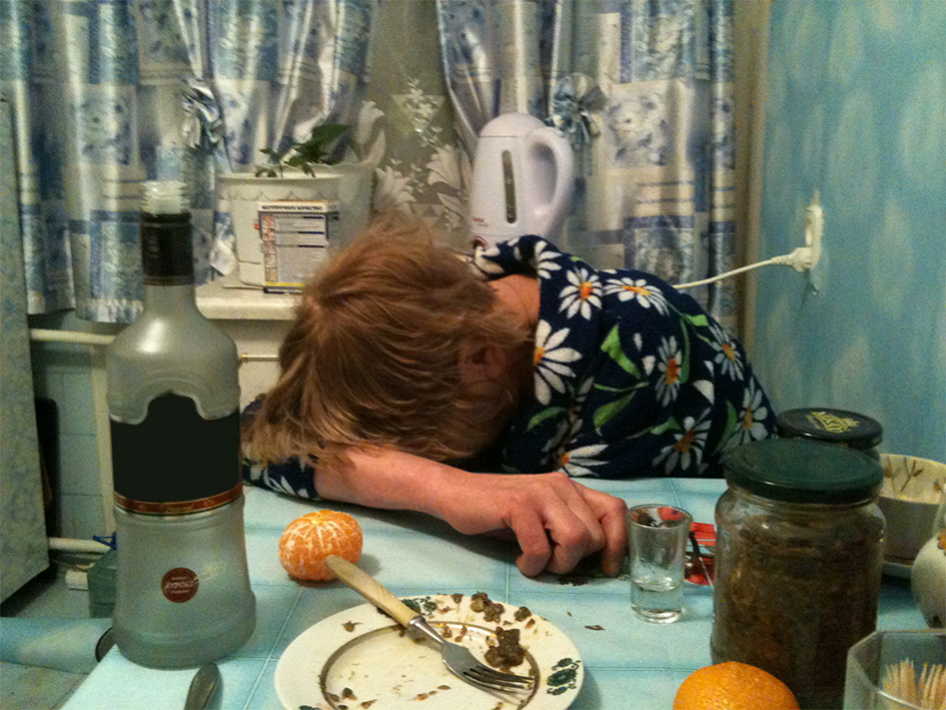
Illustration to the Case 3.

On the first sight, this case does not look like parricide. A widow aged 54 lived with her adult son in a 2-room apartment. The son treated his mother rather harshly, sometimes battered her, opened her correspondence, and forbade her using a computer: "You will make a mess!" He said that if he would not look after her, she would drink alcohol and behave immorally. In fact, however, there are physical and emotional abuses that obviously move her towards alcohol misuse and chain-smoking; she was emaciated (Figure 1) and suffered from chronic bronchitis, complicated thereafter by pneumonia; she often fell down in the street slipping on ice and already had several fractures. The social environment showed no reaction. 

## 5. Discussion and Conclusions

A concluding point is that for more successful prevention of parricide, it should be kept devoid of its reputation as an unusual horrific crime, committed by mentally ill individuals. Parricide can have trifling appearance, sometimes hardly realized as such by the victims and social environment. Perpetrators can be mentally healthy or have a personality disorder. Anger, discussed in connection with parricide (6, 15), can be absent in the perpetrators but present in the victims maltreated by their family members. The health care and social workers should take it into account and adequately react to abuse and neglect of elderly people (16).

Parricide and geronticide were practiced in the pre-historic time and in some primitive and traditional societies (17, 18) including Russian villages (19), although it can be encountered in any society. The attitude to old people, unable to work was personified by the evil characters of the folklore: Baba Yaga and Koschei the deathless, who had distinctly senile appearance. The health care altitude for the elderly in Russia is not perfect even today; in particular, middle-aged and elderly men are visibly unwelcome at the state policlinics, which is probably one of the reasons for relatively short life expectancy (20). In 2008, the difference in life expectancy between men in some West-European countries and Russia was 20 years (21). Some related aspects were commented previously (22). It should be mentioned in conclusion that most studies on parricide, elder abuse, and neglect have been based on research performed in more open societies while elsewhere it can persist without much publicity. Instructive publications addressed to the broad public are especially important (23).
